# CK2—An Emerging Target for Neurological and Psychiatric Disorders

**DOI:** 10.3390/ph10010007

**Published:** 2017-01-05

**Authors:** Julia Castello, Andre Ragnauth, Eitan Friedman, Heike Rebholz

**Affiliations:** 1 Department of Physiology, Pharmacology and Neuroscience, City University of New York School of Medicine, New York, NY 10031, USA; julia.csaval@gmail.com (J.C.); Andre.Ragnauth@gmail.com (A.R.); Friedman@med.cuny.edu (E.F.); 2 Ph.D. Programs in Biochemistry and Biology, The Graduate Center, City University of New York, New York, NY 10031, USA; 3 Ph.D. Programs at Queens College, City University of New York, New York, NY 11367, USA

**Keywords:** CK2, neurodegeneration, synapse, signaling, CK2 inhibitors, GPCRs, CK2 substrates, CK2 knockout

## Abstract

Protein kinase CK2 has received a surge of attention in recent years due to the evidence of its overexpression in a variety of solid tumors and multiple myelomas as well as its participation in cell survival pathways. CK2 is also upregulated in the most prevalent and aggressive cancer of brain tissue, glioblastoma multiforme, and in preclinical models, pharmacological inhibition of the kinase has proven successful in reducing tumor size and animal mortality. CK2 is highly expressed in the mammalian brain and has many bona fide substrates that are crucial in neuronal or glial homeostasis and signaling processes across synapses. Full and conditional CK2 knockout mice have further elucidated the importance of CK2 in brain development, neuronal activity, and behavior. This review will discuss recent advances in the field that point to CK2 as a regulator of neuronal functions and as a potential novel target to treat neurological and psychiatric disorders.

## 1. Introduction

CK2 (formerly Casein Kinase 2) is a heterotetrameric kinase consisting of two catalytic (α or α′) and two regulatory β subunits. It is constitutively active and ubiquitously expressed.

While in some instances several growth factors and neurotrophins have been shown to boost CK2 activity [1,2,3], the general consensus to date is that CK2 activity is dependent on its microenvironment within multimolecular complexes which can confer substrate specificity [4] or binding and recruitment to activity-regulated proteins such as, for example, Calmodulin [5,6].

CK2 partakes in signal transduction pathways that are crucial for cell survival such as the AKT pathway. It was shown to directly phosphorylate AKT1 (Ser129) to render AKT more active [7]. CK2 also phosphorylates and inactivates the lipid phosphatase PTEN which, by dephosphorylating phosphatidylinositol-3,4,5-trisphosphate (PIP3), reverses the activation of the AKT pathway [8]. Several other modulators of the AKT pathway are also substrates of CK2, making this kinase, clearly, an influential activator of the survival pathway. Studies have demonstrated that overexpression of CK2 potentiates tumor growth through suppression of apoptosis, promotion of angiogenesis, and signaling through PI3K-AKT, Wnt, and NF-kB [9]. Cancer cells and tissue were also shown to undergo necrosis in the presence of CK2 inhibitor [10].

Based on the minimal substrate consensus sequence that requires an acidic residue at position n + 3 [11,12], it is not too surprising that, to date, 356 proteins have been identified that are phosphorylated in vitro and in vivo by CK2 [13] and, thus, represent a large pool of bona fide substrates, attesting to the important role of CK2 as a multifunctional kinase.

In this review, we focus on recent advances in the characterization of brain-derived CK2 substrates that have been studied in an in vivo context and may have an impact in neurodegenerative disorders. We will discuss these substrates first in their roles in the healthy brain, followed by a section on CK2 in the context of brain disorders, and we point out several instances in which CK2 might prove a valid novel target for the treatment of neurological and neuropsychiatric disorders.

## 2. CK2 Expression in the Adult Mouse Brain

**Figure 1:**
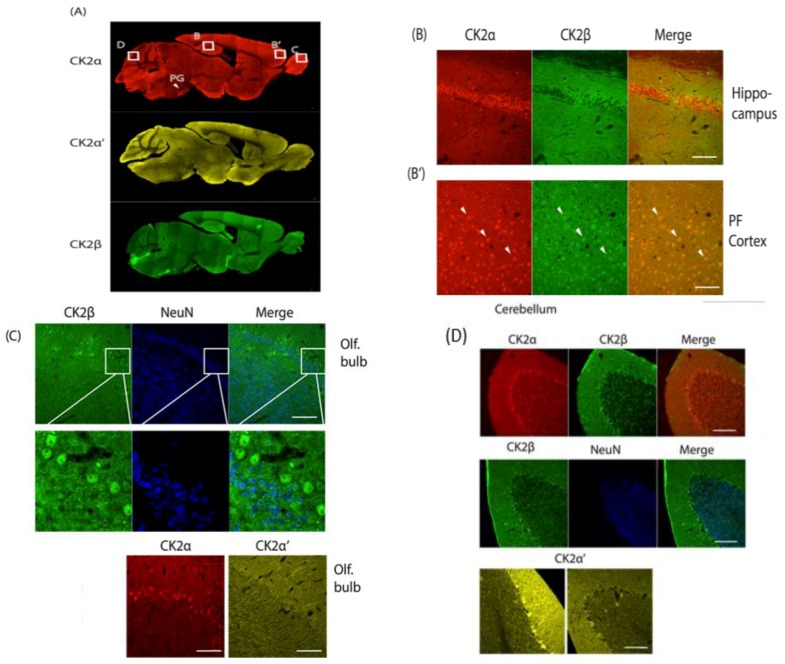
A–D: Immunohistochemical analysis of sagittal brain slices of adult C57BL/6 mice. PFA perfused brains were sliced (40 µm) and incubated with α-CK2α, α-CK2α′ (both Abcam), α-CK2β (gift from Dr. O. Filhol Grenoble) and α-NeuN (Cell Signaling) followed by incubation with secondary antibodies, Alexa 546/488 α-rabbit/α-mouse (Fisher Scientific). Imaging was performed using a Zeiss LSM710 laser-scanning confocal microscope. Slices were stained for CK2α, CK2α′ and CK2β (**A**), the hippocampal CA1region and PFC were stained for CK2α and CK2β (**B**). Olfactory bulb was stained for CK2β and NeuN, CK2α and CK2α′. (**C**) Cerebellum was stained for CK2α, CK2 β and NeuN (**D**) and for CK2α′. PG: Pontine gray nucleus; white bars = 100 μM.

CK2 is ubiquitously expressed in the periphery, and this is true for the brain as well. However, differences in expression in subregions of the brain, both on the transcript level, as can be derived from the Allen Brain Atlas, as well as on the level of protein have been observed [14,15]. Discerning the expression pattern of the individual subunits, CK2β mRNA levels do not appear balanced to the amounts of the combined CK2α and CK2α′ expression, but reflects CKα′ expression more closely. On the protein level, throughout the brain, there is a predominance of α over α′ subunits [14]. For example, in mouse striatum, a region of particular interest due to its role in movement control and reward processing, the molar ratio of CK2α:CK2α′ is 8:1. Even in the region with the highest CK2α′ protein level (hippocampus), the ratio of CK2α:CK2α′ (4:1) favors CK2α. Now, we are adding to the in situ hybridization and western blotting data immunohistochemical analyses of CK2α, α′ and β in adult mouse sagittal slices. These images depict protein expression at a much more detailed level than western blotting analysis, which is limited by the manual dissection technique. Overall, mRNA levels correspond to protein levels of the different subunits across brain regions (Figure 1A), however, some interesting patterns could be detected.

In the hippocampus, CK2α and CK2α′ proteins are present, and protein expression corresponds to mRNA levels. For CK2β, mRNA levels are the highest in the hippocampus when compared to other brain regions, but at the protein level, CK2β appears absent in dentate gyrus or the CA1, CA2, CA3 regions (Figure 1A, CA2 in higher resolution in Figure 1B). In most other regions, CK2β overlaps fully with CK2α, and we show here the prefrontal cortex as a representative example (Figure 1B’).

We found high expression of the three CK2 subunits in the pontine gray nucleus (PG), a part of the brain stem regulating motor function (Figure 1A). This area, however, is also characterized by high cell density (as visualized with a neuron-specific marker NeuN which is present in all neurons with the exception of cerebellar Purkinje cells, olfactory mitral cells, Cajal-Retzius cells, neurons of the inferior olive, and a few others) that causes the elevated immunosignal [16].

In the olfactory bulb, CK2α is very highly expressed, and, thus, the ratio of CK2α:CK2α′ is elevated to 24:1 [14]. Immunohistochemistry confirms this result. On cellular resolution, CK2β and CK2α co-localize, while CK2α′ is not detected despite significant mRNA levels in that region (Figure 1C). CK2β was counterstained with NeuN; the neuronal marker is absent from the CK2β expressing cells, and by deduction also from CK2α expressing cells. These cells supposedly are olfactory mitral cells which cannot be stained with NeuN [16] (Figure 1C).

When examining the cerebellum, we found CK2α and CK2β to be homogenously present in the large GABAergic Purkinje cells, while CK2α′ is expressed in a scattered manner. All isoforms are sporadically present in the molecular and granular layers of the cerebellar folium (Figure 1D).

The fact that no brain region was found where the CK2α′ level dominates does not imply that CK2α′ does not have a specialized vital function in the brain such as the binding to specific partners or localization to specific microdomains or subcellular structures. In fact, for example, the clustered pattern seen in the Purkinje cells of the cerebellum may be of physiological importance.

It will be interesting to take a closer look at the expression profile on a cellular level, including the use of glial markers and assess the differences in subunit expression in different cell types.

## 3. Inhibitors

A multitude of CK2 inhibitors are available, with varying specificities and efficacies [17,18]. Determining the various roles of CK2 in the brain has been difficult in part because of the poor permeability of chemical CK2 inhibitors into the brain due to the blood brain barrier (BBB). This interface separates the brain from the circulatory system, protects it from the influx of potentially harmful chemicals or organisms, and at the same time enables the transport of essential molecules bi-directionally. It is comprised of endothelial cells as well as astrocytes and pericytes adjacent to small capillaries. The high level of expression of efflux transporters (such as P-glycoprotein) in the BBB further prevents many molecules from reaching the brain. Drugs that can diffuse through the BBB must be of small molecular size (less than 500 Da), highly lipophilic, and not ionized at physiological pH [19]. This is not the case for the more specific CK2 inhibitors such as DMAT, TBB, and CX4945 which are relatively hydrophilic.

Thus far, however, one CK2 inhibitor has proven successful in reducing the growth of glioblastoma implanted intracranially in mice [20]. Mice were administered orally with CX4945, the sole CK2 inhibitor to date that has been proven to be safe and efficient in a clinical phase I/II trial in humans (ClinicalTrials.gov Identifier: NCT02128282). It has been shown that glioblastoma hamper the integrity of the BBB and this may have enabled the systemically administrated CX4945 to reach the brain tumor in sufficient quantities [21].

In our laboratory, we have performed dose and time course experiments using intraperitoneal (i.p.) injections of CX4945 in adult mice, followed by Western blotting assessment of several CK2 phosphorylation sites or of sites within the AKT pathway. At doses lower or equal 60 mg/kg, no significant reduction of these sites in striatal and prefrontocortical tissue could be detected, however, at a relatively high dose of 75 mg/kg, 2 h post-injection, the direct pS129 Akt site as well as the pS473 AKT and pS235/236 S6 ribosomal protein sites were reliably reduced, indicating that at this dose in healthy adult mice, with an intact BBB, the compound reached the brain (unpublished data). However, i.p. injection of this dose is not advisable when one aims to study CK2-dependency of certain behaviors since the mice become somewhat rigid, which indicates peripheral or central side effects that need to be further studied. The more preferable means of administration is to infuse the drug via implanted cannulae, allowing for intracranial infusion without the need of concurrent anesthesia.

Bi-substrate CK2 inhibitors, interacting with both the adenosine triphosphate (ATP) and the phospho-acceptor substrate-binding sites have been the latest addition to the collection of CK2 inhibitors. While highly effective [22], thus far, these types of compounds are unable to penetrate cells or the BBB.

New strategies are being developed to overcome the restraints of the BBB, such as the use of liposomal, magnetic, or polymeric nanoparticles that are either coated or conjugated with targeting moieties that enable carrier-, receptor-, and absorption-mediated passage through the BBB (for a review see [19]).

One such example for CK2 is the successful delivery of tenfibgen nanocapsules with a siRNA CK2 cargo via intravenous administration, which resulted in significant xenograft tumor reduction in a mouse model of prostate cancer [23]. Recently, a dual approach was tested: CK2 and epidermal growth factor receptor (EGFR), both of which are overexpressed in glioblastoma [24], were targeted by morpholino oligomeres attached to nanobioconjugates [25], in a mouse model of intracranial human glioblastoma. This lead to increased animal survival and concomitantly reduced pro-survival (AKT, STAT, Wnt) signaling. The nanoconjugates were linked to anti-tranferrin antibody that enabled BBB passage and an anti-EGFR antibody to attach to cancer cells [25]. One could envisage the packaging of CK2 inhibitors into nanocapsules as well.

## 4. CK2 Knockout Mice

Knockout (KO) mice are in many ways superior to inhibitors when one wants to detect physiological functions of a specific gene product since they allow for deletion or insertion of a specific gene through homologous recombination, thus, not affecting other genes. However, one has to be aware that developmental adaptations may occur, especially in full knockout or conditional models with early embryonic Cre recombinase expression. KO mice have clearly shaped our understanding of the crucial role of CK2 in development: full CK2β knockout mice are embryonic lethal. The embryos are absorbed on embryonic day E7.5 [26]. Conditional CK2β KO mice were generated and crossed with a Nestin-Cre driver to generate mice deficient in CK2β in the central nervous system, but mutant pups died shortly after birth [27]. Interestingly, the CK2β protein was detected in KO mice six days after onset of Cre expression, which could be rationalized by the long stability of the CK2β protein once integrated in the CK2 holoenzyme. It is interesting to note that CK2α expression was not altered in the KO mice [27].

A full CK2α KO mouse line was also generated: while the heterozygous offspring are born at the Mendelian ratio, the homozygous embryos die mid-gestation (E11.5) [28]. The strongest defects were seen in the heart and neural tube, pointing towards an important role of CK2 in brain and heart development. Interestingly, expression of CK2β is reduced while CK2α′ expression is unaffected in the CK2α KO [28,29].

CK2α′ KO mice are viable and do not exhibit an obvious phenotype. Only homozygous males are infertile because spermatocytes frequently undergo apoptosis or have abnormal heads (as in the human condition globozoospermia) [30]. It is of interest to note that due to the lack of apoptosis-positive cells (TUNEL assay) in the CK2α and CK2β KO embryos, it was suggested that, during embryonic development, CK2α and CK2β are mainly controlling cell proliferation and not apoptotic events [29].

To study the role of CK2 in the brain, we generated CK2α^fl/fl^ and CK2α′^fl/fl^ lines. Mice lacking CK2α′ in the forebrain (CK2α′^fl/fl^; CaMKII-Cre) were viable and did not display an obvious behavioral or pathological phenotype. In contrast, neither the CK2α^fl/fl^; CaMKII-Cre or CK2α^fl/fl^; CK2α′^fl/fl^; CaMKII-Cre were viable, the mice died perinatally [14]. CaMKII-Cre has been described as readily detectable at postnatal day three [31], however, due to the perinatal death of our offspring, we suspect that sub-threshold expression already occurs days earlier. The non-viability of the Cre-positive offspring indicates that a short depletion of CK2 activity at the developmental stages around birth is crucial for viability. 

We also generated cell-specific neuronal KO mice by crossing the CK2α floxed animals with the Drd1a-Cre or the Drd2-Cre driver lines which leads to ablation of CK2α in the dopamine D1 receptor expressing cells of the striatum and cortex, and in the D2 receptor expressing cells of the striatum and midbrain, respectively. Notably, the double KOs, homozygous for CK2α and α′ deletion, were not viable with any of the Cre-driver lines tested. However, the single KOs homozygous for CK2α or α′ isoforms were viable with both the Drd1a-Cre and the Drd2-Cre driver mouse lines [32]. Cre expression starts at E16 for the Drd1a-Cre and at E14 for Drd2-Cre [33,34]. Interestingly, expression of the β subunit was not affected in the Drd1a-Cre-CK2α KO while it was significantly reduced in Drd2-Cre CK2 KO.

The Drd1a-Cre CK2 KO mice exhibited distinct behavioral phenotypes including novelty-induced hyperlocomotion and exploratory behavior, defective motor control, and motor learning. These traits are indicative of dysregulated dopamine signaling and were rescued by dopamine D1 receptor antagonist [32]. The underlying mechanisms involved in the phenotype are probably manifold: in addition to altered D1 receptor homeostasis, altered synaptic activity may be involved, since, as will be discussed in the next paragraph, CK2 was shown to regulate several glutamate receptors [5,6,35].

The importance of CK2 in the dopaminergic system is further highlighted by preliminary findings indicating that in a mouse model of Parkinson’s disease, a response to the antiparkinsonian drug, L-DOPA, namely uncontrollable dyskinesia, is affected in KO mice (unpublished data).

The floxed CK2α, CK2α′ and CK2β mouse lines are amenable to focal knockdown of CK2 since Adeno-associated virus (AAV) or Lentivirus can be used to induce Cre recombinase in small to more widespread areas around an injection site, depending on the viral serotype. This approach will certainly be valuable to address whether a specific brain region mediates certain phenotypes.

## 5. Brain Specific Substrates

CK2 is a pleiotropic kinase with several hundred potential substrates [36]. Many substrates are not only important in the periphery but have similar roles in the brain. An extensive review of such substrates can be found in [37]. In this section, we aim to discuss in vivo substrates of CK2 that are either specific to the brain or are crucial for healthy brain function. Many of these candidates are also involved in brain disorders and suggest that CK2 could be a valid novel target for pharmacotherapy for such disorders, as will be discussed in Section 6 of this review.

### 5.1. G Protein Coupled Receptors (GPCRs)

G protein coupled receptors (GPCRs) make up the largest protein family in the human genome and consist of over 800 members [38]. These receptors mediate the biological actions of neurotransmitters, hormones, pheromones, light, and calcium through the activation of one or more of the four G protein families: Gα_i/o_, Gα_q/11_, Gα_s_, and Gα_12/13_. GPCR cell surface expression and coupling to G-proteins are regulated by phosphorylation of their third intracellular loop and/or their C-terminal region by various Ser-Thr kinases, such as G protein-coupled receptor kinases (GRKs1–7) [39]. Binding of arrestins to phosphorylated receptors results in uncoupling of the receptor, desensitization of the response, and endocytosis of the receptor [39,40]. Second messenger-dependent protein kinases, such as protein kinase A and protein kinase C, or CK1α, are also involved in GPCR desensitization [41]. More recently, CK2 joined the pool of kinases that are capable of phosphorylating GPCRs. Torrecilla et al. showed that the muscarinic M3 acetylcholine receptor is phosphorylated by CK2 in the third intracellular loop upon agonist occupation [42]. However, in this case, no internalization except a signaling switch to a Jun-kinase dependent pathway is induced. In pancreatic ß-cells, CK2 was shown to phosphorylate the same receptor, M3 [43], with the effect of reduced insulin release. Both, CK2 inhibition and knockdown of CK2α in β-cells resulted in M3 receptor-stimulated insulin release. Again, in this case, phosphorylation did not affect receptor internalization or signaling. These two papers demonstrate that CK2 is capable of affecting the same receptor, in different cell types, resulting in different outcomes. The determination as to which outcome phosphorylation has most probably depends on the expression of tissue-specific proteins and/or on the specific phosphorylation site.

A different involvement of CK2 in the regulation of GPCRs in the brain was identified following a yeast-two-hybrid screen which yielded the G protein subunit Gα_s_ as a CK2β interacting partner in cultured cells and in brain tissue. The complex also contained CK2α, indicating that the CK2 holoenzyme is bound to Gα_s_ [44]. The interaction was specific to Gα_s_ since no other Gα subunit precipitated with CK2β. Functionally, this interaction suggests negative regulation by CK2 of Gα_s_ signaling since CK2 inhibition or siRNA targeting CK2α reduced agonist-induced receptor endocytosis in cultured cells and concomitantly enhanced receptor signaling. The regulatory effect of CK2 was also observed for the Gα_s_-coupled adenosine A2a receptor [44]. The identity of the substrate for CK2 that is involved in the regulation of Gα_s_-coupled receptor signaling is currently unknown.

The implication of the above studies is that CK2 has the potential to modulate a whole set of GPCRs. It is estimated that roughly 15% of 170 well-studied non-olfactory GPCRs signal via Gα_s_ [45]. Many of these GPCRs are expressed in the brain and are important pharmacological targets involved in a variety of neurological disorders. For example, major depressive disorder, affecting up to 1 in 5 adults in the USA [46] is related to dysfunction in brain serotonergic system. Three of the 14 serotonin receptor subtypes are Gα_s_-linked and are, therefore, candidates for regulation by CK2. We have preliminary evidence showing that one of these serotonin receptors, the 5-HT4 receptor, is regulated by CK2 (unpublished data).

Other neurological diseases in which Gα_s_ coupled receptors play major roles are Parkinson’s disease (PD) which is characterized by a hypersensitization of the Gα_s_-coupled dopamine D1 receptor. In PD, adenosine A2a receptors control the activity of neurons that oppose the action of the D1 receptor. A2a antagonists have been shown to exert potent anti-akinetic effects in animal models of PD and are currently being evaluated in clinical trials [47]. One could, therefore, hypothesize that modulation of CK2 could have beneficial effects via regulation of both D1 and A2a receptors in Parkinson‘s disease.

### 5.2. CK2 Substrates Involved in Synaptic Transmission

CK2 is present in the nucleus and cytoplasm of neurons, but it is also clearly localized at the plasma membrane [44], and it is accumulated at the post-synaptic density in rat hippocampal and cortical preparations [48]. In vitro, PSD-95 was shown to be a CK2 substrate [48]. CK2 was further shown to co-localize with the *N*-methyl-d-aspartate receptors (NMDAR) subunit NR1 at the synapse [5]. Finally, CK2 activity was found to be enriched in synaptosomes [15].

Work of several groups has highlighted the importance of CK2 in the regulation of the ionotropic glutamate receptors α-Amino-3-hydroxy-5-methyl-4-isoxazolepropionic acid (AMPA) and NMDAR. With glutamate being the major excitatory neurotransmitter, it is logical that modulation of these receptors impacts neuronal excitability and synaptic transmission. Both glutamate receptor types are CK2 substrates, and their activity is modulated by CK2. For example, pharmacological inhibition of CK2 reduced NMDAR activity [49]. The NR2B subunit of NMDAR is phosphorylated in vitro and in vivo by CK2, which leads to a disruption of receptor interaction with PSD-95 and a reduced cell surface expression of the receptor [5]. This internalization process was a response to receptor activation, suggesting that CK2 is involved in receptor desensitization. Since CK2 is known as a constitutively active kinase, the question was how this subunit internalization may be regulated in an activity-dependent manner. It was shown that the Ca^2+^/calmodulin-dependent protein kinase II (CaMKII), activated through activity-induced calcium influx, recruits CK2 into a trimeric complex together with NR2B. A NR2B mutant that cannot bind to CaMKII is less phosphorylated at the CK2 site (S1480) and has increased surface expression [6].

While the NR2A subunit of the NMDA receptor is not a CK2 substrate, is it still indirectly regulated by CK2 since phosphorylation-dependent NR2B-endocytosis results in an increase in synaptic NR2A expression. It was shown that this switch from NR2B to NR2A is crucial and corresponds to a surge in CK2 expression during embryonic development and, as was later also shown in hippocampal neurons, was dependent on NMDA receptor activity [35,50]. This switch from a NR2B to NR2A subunit in a CK2 dependent manner, was also detected in adult brain hypothalamic neurons, resulting in increased neuronal excitability [51]. The NMDA activity-dependent action of CK2 should be seen separately from the action of CK2 on activating the receptor, which was proposed to be either mediated through a different phosphorylation site or an indirect mechanism [49].

Recently, a role for CK2 in the regulation of cell surface expression of the AMPA receptor subunit GluA1 was proposed in cultured hippocampal cells; in such a scenario, phosphorylation by CK2 leads cell surface accumulation of GluA1 as opposed to internalization of the NR2B subunit of the NMDAR [52].

Glutamate receptors are not the only molecules involved in synaptic function that are CK2 substrates; others are, for example, synaptotagmin, a transmembrane protein involved in the synaptic vesicle fusion with the presynaptic membrane [53], syntaxin, a synaptotagmin interacting protein [54], and dynamin 1, a microtubule stimulated GTPase involved in endocytosis [53,55,56]. However, these phosphorylation events have not, as yet, been detected in vivo.

Another family of membrane proteins that modulate synaptic activity are the voltage gated sodium channels (NAvs). Recently, the CK2 inhibitor TBB was shown to reduce excitability of neurons by abolishing CK2-mediated phosphorylation of the fibroblast growth factor receptor FGF14 and reduced interaction of FGF14 with voltage gated sodium channels (NAv1.2 and 1.6) [57]. CK2 was further found to directly phosphorylate the voltage gated sodium channel NAv1, thereby enhancing its binding to ankyrin and accumulation at the axon initial element, an event which is necessary for fast propagation of action potentials [58]. Small conductance Calcium-activated K+ (SK) channels are gated by the Ca^2+^ sensor calmodulin. Phosphorylation of calmodulin by CK2 reduces its Ca^2+^ sensitivity and leads to channel deactivation in xenopus oocytes [59].

Molecules involved in slow synaptic transmission are also phosphorylated in vitro by CK2. These include the phosphatases PP2a and PP2c and the kinases PKA and PKC, to mention just a few. For an extensive review on signaling proteins that are CK2 substrates, please refer to [3,36]. One example of an in vivo CK2 substrate that links cyclic AMP (cAMP) signaling to nuclear responses, changes histone phosphorylation and transcription is the dopamine- and cAMP-regulated neuronal phosphoprotein (DARPP-32), a regulator of the phosphatase PP1 that is highly expressed in striatum [60]. CK2 phosphorylation of DARPP-32 enhances its potency to inhibit PP1 [61,62]. DARPP-32’s translocation to the nucleus, where it controls histone H3 phosphorylation and transcriptional activation, depends on the phosphorylation state at the CK2 site. Mutation of the CK2 phosphorylation site alters the behavioral effects of drugs of abuse and decreases motivation for food [63]. 

### 5.3. Substrates Involved in Proteostasis

Many neurodegenerative disorders originate from protein misfolding processes. Examples are Huntington’s disease, spinocerebellar ataxia, Parkinson’s and Alzheimer’s diseases. The presence of cellular defense mechanisms like molecular chaperones and proteasome degradation systems prevent protein misfolding and aggregation, but these systems may not respond sufficiently under conditions of permanently elevated levels of proteins with high aggregation propensities. Mis- or unfolded proteins first aggregate to soluble oligomers, then to insoluble amyloid fibrils, which are structurally defined by β strands. The capacity of protein quality control and degradation (proteostasis) declines with aging, facilitating neurodegeneration as exemplified by the late onset of excessive accumulation of amyloid-beta peptide (Aβ) and tau in Alzheimer’s disease, and α-synuclein in Parkinson’s disease [64]. Chaperones have been shown to interfere at various steps of the aggregation cascade including nucleation and fibril elongation and are members of diverse signaling pathways, including the heat shock response activated after acute stress, the ubiquitin-proteasome system, and the autophagosome-lysosome pathway [65,66]. While the aggregated protein itself, often inherited in a mutated version, differs for each disease, the pathways controlling proteostasis are overarching mechanisms of which CK2 has been shown to regulate several major players. 

CK2 phosphorylates and modulates several chaperones such as Hsp90 as well as its co-chaperones FKBP51, 52 and Cdc37. CK2-dependent phosphorylation of Cdc37 is essential for the chaperone function of Hsp90-Cdc37 [67]. FKBP51 and 52 regulate, among others, steroid hormone receptor activity: FKBP52 activates while FKBP51 inactivates these types of receptors. Phosphorylation by CK2 completely abrogates FKBP52 regulation of receptor function, thus, leading to a reduction in receptor activation. FKBP51 and FKBP52 are also discussed, together with Hsp90, for their impact on tau phosphorylation and stability. Again, there seems to be an indication that both co-chaperones act in opposing ways, with FKBP51 stabilizing tau and FKBP52 reducing tau stability [68]. To date, these questions are not entirely resolved.

CK2 was shown to phosphorylate Hsf1, leading to nuclear accumulation of Hsf1 in vitro [69], while mutation of phosphosites to alanine inhibited the transcriptional activity of Hsf1. This finding could be of great interest since Hsf1 is the regulator of various chaperones, including Hsp70. 

Enhancing the activity of the ubiquitin-proteasome pathway is a promising strategy to ameliorate protein aggregation diseases. In this context, CK2 was found to modulate the expression of proteasome genes via Nrf1 phosphorylation. Knockdown of CK2 enhances the Nrf1-dependent expression of proteasome subunit genes and reduces the accumulation of ubiquitylated proteins in vitro in several cell lines [70]. Like several other kinases, CK2 has been shown to target proteins for degradation through ubiquitin-mediated proteolysis [71]. 

Taken together, like in other instances, CK2 has a bidirectional effect on the proteasome: phosphorylation of certain substrates enhances their proteasomal degradation, while phosphorylation of transcription factor Nrf1 reduces its activity to mediate expression of members of the proteasome family. It is conceivable that the effect of Nrf1 on the synthesis of proteasome components and, thus, on overall proteasome output, may outweigh the role of CK2 on targeting a handful of substrates to the proteasome. However, one would need to integrate these data, as well as the effects on the proteasome and on heat shock response, to estimate the net effect of CK2 on protein aggregation in several systems, in cell culture, and, ideally, in preclinical models.

## 6. Diseases of the Human Brain

### 6.1. Glioblastoma

Glioblastoma multiforme (GBM) comprise 15% of all brain tumors and are the most aggressive human glial tumors with a median survival of 14–15 months [72]. Unfortunately, tumors often become drug-resistant and regrow despite chemotherapy. CK2α is overexpressed in human GBM; this is caused by a gain in gene dosage of approximately 34% [20], while a 4-fold increase in CK2α protein is measured [73].

Inhibition of CK2 activity through small molecule inhibitors or siRNA induced apoptosis, reduced growth in GBM cells in mouse xenograft models of human GBMs [20] as well as in mice that had been injected intracranially with human GBM tumors. Concomitantly, CK2 inhibition lowered JAK/STAT (Janus kinase/signal transducers and activators of transcription pathway) and NFkB activation as well as the activation of survival markers of the AKT pathway and promoted survival of mice [20,25,74]. These data are very promising and clearly warrant clinical trials.

For the remainder of this section, we would like to focus on diseases where not proliferation but rather cellular degeneration occurs, namely neurodegenerative diseases such as Parkinson’s and Huntington’s diseases, Amyotrophic lateral sclerosis, and Alzheimer’s disease.

### 6.2. Parkinson’s Disease

Parkinson’s disease (PD) is the second most prevalent neurodegenerative disorder, affecting as many as 2% of people 65 years or older [75]. It is characterized by symptoms such as tremor, rigidity, bradykinesia, and gait disturbances. The major cytopathological markers of the disease are the loss of dopaminergic neurons in the midbrain and the formation of Lewy bodies, mainly composed of aggregated α-synuclein fibrils, in the remaining dopaminergic neurons [76]. Phosphorylation of α-synuclein at Ser-129, close to its C-terminus is held as a hallmark of Parkinson’s disease mainly because α-synuclein within Lewy bodies is extensively phosphorylated at this site. It was shown that CK2 as well as polo-like kinase (PLK) phosphorylate soluble α-synuclein at S129, while PLKs are most probably responsible for phosphorylation of aggregated α-synuclein [77]. Overexpression of PLK but not CK2 in cultured cells increased phosphorylation of aggregated α-synuclein at S129 [78]. By employing a series of mutated substrates, in vitro experiments by Salvi et al. demonstrate that the polo-like kinases PLK2 and PLK3 are more efficient at phosphorylating α-synuclein at this site than CK2 [79]. Indeed, using striatal tissue from conditional CK2 KO mice (Drd1-Cre or Drd2-Cre) we could not detect a reduction in pS129 α-synuclein (unpublished data) despite a 50% loss of CK2α protein (and no compensatory upregulation of CK2α′). Thus, one can conclude that while CK2 is an efficient in vitro kinase for this site, it is not the major kinase that performs this phosphorylation in vivo. The physiological relevance of this phosphorylation is still heavily debated since experiments in rats, mice, and drosophila as well as in tissue culture lead to opposing results and do not allow the conclusion that pS129 a-synuclein is causal to the aggregation or to toxic effects [80]. 

Another protein that is localized to Lewy bodies and binds to α-synuclein is synphilin. It has been reported that, in vitro, CK2 phosphorylates synphilin and thereby alters the interaction to α-synuclein [81].

Based on our work with CK2 KO mice, we hypothesize that in addition to the potential CK2 substrates described above, CK2 may have a central role in modulating the brain’s responses to dopamine depletion as well as to anti-parkinsonian treatment. We have shown that the activity of two major dopamine responsive neuronal cell types in the striatum, namely the direct and indirect pathway spiny projection neurons, is modulated by changes in expression and plasma membrane availability of the dopamine D1 and the adenosine A2a receptors, respectively [32,44] Both these receptors are primarily responsible for behavioral responses to dopamine and L-DOPA, therefore, we presume CK2 must have a role in Parkinson’s disease; this investigation is currently under way. 

### 6.3. Huntington’s Disease

Huntington’s disease (HD) is a progressive neurodegenerative disorder mainly affecting medium spiny neurons of the striatum. It is the consequence of an expansion of the CAG sequence in the huntingtin gene that causes the translation of an expanded polyQ tail in the protein. The symptoms are categorized into motor and cognitive in nature, such as frontal lobe dementia, and psychiatric symptoms, including depression, anxiety, and psychosis [82]. Fan et al. have published in vitro evidence that cells overexpressing polyQ-huntingtin, exhibit enhanced CK2 expression [83]. This observation was validated in a mouse model of HD where upregulation of CK2 takes place in the striatum but not in the cortex. When mice were treated with a pharmacological CK2 inhibitor, there was a significant increase in NDMAR mediated toxicity which could be explained by the phosphorylation-induced endocytosis of NMDAR [5]. Taken together, the possible role of CK2 is to counteract the toxic effects of the mutant huntingtin gene on NMDAR activity.

One example of a second polyQ disease with pathophysiological involvement of CK2 is spinocerebellar ataxia type 3. CK2-mediated phosphorylation leads to nuclear accumulation and aggregation of ataxin 3, indicating that, in the context of this disease, inhibition of CK2 could have the desirable effect of reducing its nuclear aggregations [84].

### 6.4. Amyotrophic Lateral Sclerosis

Amyotrophic lateral sclerosis (ALS) is a neurodegenerative disorder that involves the loss of motor neurons of the cortex, brainstem, and spinal cord leading to paralysis and, ultimately, death by respiratory failure [85]. As in other neurodegenerative diseases, protein aggregates are detected in the affected areas of the brains of patients, although the aggregated protein may vary in different forms of the disease. The nuclear RNA-binding protein TDP-43 is, however, found in cytoplasmic inclusions of all forms of ALS (except in cases of familial ALS with mutations in SOD1).

TDP-43, trapped in the aggregates, undergoes ubiquitination and hyperphosphorylation, and CK2 was found to drive phosphorylation at various sites in cultured cells. Overexpression of CK2 increases phosphorylation, facilitating the solubility of TDP-43 truncated mutants, and this effect is reversed when cells are treated with a CK2 inhibitor. A Drosophila mutant line expressing a non-phosphorylatable point mutant developed aggregates in neurons, while a phosphomimicking mutant failed to do so. These studies suggest that CK2 upregulation and enhanced phosphorylation of TDP-43 may prevent TDP-43 aggregation and be beneficial for disease outcome [86].

### 6.5. Alzheimer’s Disease

Late-onset Alzheimer’s disease (AD) is the most common neurodegenerative disorder in the USA and Europe and is characterized by progressive worsening in cognitive functions along with functional and behavioral impairments. The pathophysiological hallmarks of the disease are extracellular insoluble beta amyloid (Aβ) plaques and intracellular neurofibrillary tangles (NFTs) composed of hyperphosphorylated tau aggregates. CK2 activity and protein expression were found to be reduced in Alzheimer’s disease [87]. CK2 was detected in association with neurofibrillary tangles whose main component is accumulated and hyperphosphorylated tau [88]. Tau purified from human brain and tau in neuroblastoma cells are CK2 substrates [89,90].

CK2 was found to phosphorylate Apolipoprotein-E at an atypical site involving proline and an acidic residue at +1, and interaction of these two partners rendered CK2 more active towards tau in in vitro kinase assays. However, this data still needs to be confirmed in an in vivo setting [91].

A strong body of evidence suggests that soluble amyloid-β peptide (Aβ) oligomers induce synaptic loss in AD. Aβ-induced synaptic dysfunction is dependent on overstimulation of *N*-methyl-d-aspartate receptors (NMDARs) resulting in aberrant activation of redox events as well as elevation of cytoplasmic Ca^2+^, which in turn triggers phosphorylation of tau and the activation of caspases Cdk5/dynamin-related protein 1 (Drp1) and CaMKII [92]. Dysfunction in these pathways leads to mitochondrial dysfunction, synaptic malfunction, impaired long-term potentiation, and cognitive decline. Aβ synaptic toxicity can be partially ameliorated by NMDAR antagonists (such as memantine). As described in the above section on synaptic transmission (Section 5.2), since Aβ was shown to activate CK2 in vitro [93], one could hypothesize that CK2 activation leading to enhanced prolonged NMDA channel opening [49] may be partially responsible for the excessive NMDAR toxicity.

Several laboratories also demonstrated that CK2 is linked to the processing of the amyloid precursor protein (APP). Walter et al. showed that APP is a substrate for the ectokinase CK2 [94], after it had been demonstrated by several groups that CK2 can be shed and can phosphorylate extracellular substrates [95,96]. In neuroblastoma cells, CK2 inhibitor reduced the processing of APP to soluble sAPPα in response to cholinergic stimulation [97]. sAPPα is generated by α-secretase and precludes processing of APP by β and γ-secretases. Thus, this effect caused by CK2 may be desirable, however, further experiments in this direction need to be undertaken.

As mentioned above, CK2 is activated by Aβ in vitro [93]. Such an activation was proposed to result in an inhibition of fast axonal transport (FAT), which is a mechanism by which synaptic proteins and mitochondria are transported from the cell body into axons for proper neuronal function and survival. Inhibition of CK2 rescued axonal transport and overexpression of active CK2 mimicked the inhibitory effects of Aβ on FAT. The effect of CK2 on FAT is believed to be mediated by phosphorylation of kinesin-1 light chains and subsequent release of kinesin from its cargoes, effectively disabling the transport [98].

However, this finding must be evaluated in the light of data showing that in cultured mammalian cells, reduction of CK2 expression decreases the number of active kinesin motors [99]. Thus, CK2 up-regulates kinesin-based transport by enhancing the kinesin number but also releasing kinesin from its cargoes, yielding two functions that counteract each other. Further investigation will help to resolve the question of which of the effects is predominant in vivo.

Recent human and preclinical studies have provided evidence that impaired insulin signaling and glucose utilization are contributing to the pathophysiology in AD [100]. It was shown that insulin and the insulin-sensitizing drug rosiglitazone improve cognitive performance in mouse models of AD and in patients with early AD [101,102] by reducing binding of Aβ oligomers to synapses. In contrast, patients with insulin-resistant type 2 diabetes show an increased risk of developing AD [100]. In this context, it is worthwhile to note that in response to Aβ oligomer binding to hippocampal neurons, CK2 and CaMKII were found to mediate internalization of the insulin receptor. These findings are in several ways reminiscent of CK2’s role in NDMAR endocytosis (as discussed in Section 5.2) since both require CaMKII and are dependent on receptor activity. 

Other groups have already identified a role for CK2 in the non-neuronal insulin pathway as described in Section 5.1, CK2 negatively modulates insulin release from pancreatic beta cells, in a manner that depends on the M3 receptor [43]. While this work is discussed in the realm of glucose intolerance and diabetes type 2, one could extend a hypothesis here to question if pharmacological CK2 inhibition might benefit Alzheimer’s patients.

Neuroinflammation, as detected by the presence of activated complement proteins interleukins and chemokines in microglia, and astrocytes are is increased in AD [103]. Although neuroinflammation in the brain of AD patients is considered primarily beneficial (eliminating injurious stimuli and restoring tissue integrity), a chronic neuroinflammatory response may be harmful due to the constant excess of pro-inflammatory cytokines, prostaglandins, and reactive oxygen species. A recent immunohistochemical study detected increased amounts of CK2α or α′ in the hippocampus and temporal cortex of AD patients in astrocytes surrounding amyloid deposits [104]. It remains to be determined whether this increase is of functional consequence.

In summary, CK2 plays a role in several mechanisms that are involved in tau phosphorylation, APP processing, Aβ signaling, and protective responses to Aβ insults such as neuroinflammatory responses and insulin signaling. 

## 7. Conclusions

In summary, it comes as no surprise that CK2 plays a major role in a variety of processes in the brain, as it has been shown to target a vast number of brain proteins. In the context of brain disorders, one would like to assess whether CK2 may be a good target for modifying disease outcome and or progression. 

The case for glioblastoma is clear, and several preclinical studies have demonstrated the beneficial effect of CK2 inhibitors on tumor size reduction as well as patient survival. 

For Huntington’s disease and ALS, there is currently no rationale to argue for CK2 as a target to interfere with disease progression, while in the case of type 3 spinocerebellar ataxia, CK2 inhibition may be desirable and result in a reduction in ataxin aggregation. 

In the context of Alzheimer’s disease, most known factors would argue that, even though CK2 expression was shown to be reduced, a further inhibition of CK2 through pharmacological means may be beneficial in reducing the burden of hyperphosphorylated tau and neurofibrillary tangles. It may also be beneficial to prevent or reduce insulin receptor internalization and to rescue fast axonal transport that is disabled by Aβ. However, CK2 activity may be desirable due to its endocytotic effect on NMDAR.

In order to ascertain how the various mechanisms of CK2 involvement in neurological disease pathology are integrated in vivo, it is preferable to use a conditional CK2 KO, possibly even in an inducible version, and cross the KO mice into appropriate preclinical models of neurodegenerative diseases. The genetic approach is, in our opinion, preferable for proof of concept experiments because the available inhibitors are not readily blood-brain barrier permeable and, thus, require relatively high-dose administration which will also affect peripheral sites and a variety of centrally mediated behaviors that are unrelated to disease pathologies.
